# Practice of Acceptance, Awareness, and Compassion in Caregiving (PAACC): A randomized controlled trial on mindfulness‐based intervention for caregivers of individuals living with dementia

**DOI:** 10.1002/alz70858_099166

**Published:** 2025-12-25

**Authors:** Mamta Sapra, Lauren Hagemann, Katherine Luci, Jyoti Tina Savla

**Affiliations:** ^1^ Veteran Affairs Medical Center, Salem, VA, USA; ^2^ Virginia Tech Carilion School of Medicine, Roanoke, VA, USA; ^3^ Center for Gerontology Virginia Tech, Blacksburg, VA, USA

## Abstract

**Background:**

As the prevalence of dementia is predicted to increase three‐fold in the coming decades, there is critical need for effective interventions to support caregivers who play essential role in the health and wellbeing of individuals with dementia. Current evidence‐based caregiver interventions, such as Resources for Enhancing All Caregivers Health (REACH‐VA), focus on cognitive and behavioral strategies to lessen caregiver burden. The Practice of Acceptance, Awareness, and Compassion in Caregiving (PAACC) combines mindfulness and practical caregiving skills to reduce caregiver strain and enhance caregiver's resilience. The goal of this intervention is to help caregivers develop increased present‐focused acceptance and awareness of their reactivity to care‐related stressors, while fostering empathy towards their loved one with dementia. This approach differs from strategies that challenge or replace negative or unrealistic stress‐related thoughts, considering the often progressive and unchangeable nature of stressors in dementia. This study compared the effectiveness of a mindfulness based PAACC intervention to cognitive behavioral based REACH‐VA intervention.

**Method:**

This study enrolled 133 dementia caregivers with moderate to severe levels of caregiver burden who were randomized to either cognitive behavior based (REACH‐VA) or mindfulness based (PAACC) support intervention. Study assessed caregivers for stress levels, perceived stress, rumination as well as mindfulness using validated questionnaires.

**Results:**

The mean age of caregivers was 67yrs, predominantly white and most lived with their loved one that they were caring for. Both interventions reduced perceived stress, caregiver burden, and rumination. Caregivers who received PAACC showed lower anxiety (B=‐2.76, *p* <0.001) and depressive symptoms (B=‐2.50, *p* <0.01). PAACC participants exhibited higher levels of mindfulness after the intervention, with a significant increase indicated by a coefficient of 6.04.

**Conclusion:**

The study shows that while both interventions are beneficial, PAACC offers an additional advantage in enhancing psychological well‐being of the caregivers. The increase in mindfulness suggests that PAACC caregivers were able to internalize practices taught in the intervention leading to better coping strategies. The results highlight the potential benefits of integrating mindfulness into future caregiver interventions.

